# A quantitative study exploring the acceptance of the eHealth model for mental wellness among digital workers

**DOI:** 10.12688/f1000research.73482.1

**Published:** 2022-01-28

**Authors:** Choon Hong Tan, Ah Choo Koo, Hawa Rahmat, Wei Fern Siew, Alexius Weng Onn Cheang, Elyna Amir Sharji

**Affiliations:** 1 Persiaran Multimedia, Multimedia University (MMU), Cyberjaya, Selangor, 63100, Malaysia; 2 International Medical University (IMU), 126, JIn Jalil Perkasa 19, Bukit Jalil, Kuala Lumpur, 57000, Malaysia

**Keywords:** eHealth, digital talents, mental wellness, survey, quantitative analysis

## Abstract

Background: eHealth makes use of information and communication technologies (ICT) to improve health. In the digital age, the use of eHealth applications and other health-related applications has gained popularity, particularly during the COVID-19 pandemic. As a result of the pandemic, many uncertainties have arisen, causing stress and affecting the mental health of many skilled workers in the digital industry, particularly in the ICT, computing, and creative media industries. eHealth applications have the potential to benefit people's health. As a prerequisite for effective implementation of eHealth for mental wellness (EHMW), this paper examines the acceptance of EHMW among digital workers in Malaysia.

The objectives of this research are two-fold: 1) To explore the acceptance of EHMW among digital workers in a local Premier Digital Tech Institution (PDTI), and 2) To explore how these talents' demographic profiles, mental health literacy and workplace wellness influence their acceptance of EHMW.

Methods: This research surveyed 41 digital workers who played vital roles in providing digital skills at a tertiary education level.

Results: Most respondents agreed that eHealth was appropriate for managing mental wellness. Among the three eHealth domains for managing mental wellness, the acceptance level is the highest for the application domain of "interacting for health", with male respondents more likely to accept the use of EHMW.

Conclusions: This small-scale survey could not fully examine the acceptance of eHealth and its usage patterns for mental wellness among digital workers in Malaysia. Future research will target more digital workers in Malaysia. This research addresses the research gap on the eHealth perspectives of digital workers on their acceptance, and the potential influence of demographic profiles, mental health literacy, and workplace wellness on EHMW's acceptance of digital health tools/platforms to promote their mental wellness.

## Introduction

Following the COVID-19 pandemic, increasing amounts of working adults in Malaysia are facing general health and mental health problems due to concerns such as personal finances and health problems. ^4^ Since the introduction of the Movement Control Order (MCO), most workers, especially digital workers who are working in digital industries and involved in ICT-related work areas, ^8^ have moved to working from home (WFH) using various digital technologies and platforms. Therefore, digital skills have become essential, and digital literacy has become a critical subset of the skills acquired by digital workers. WFH has the advantage of flexibility in terms of working hours and location, but there are also disadvantages, such as the blurring of boundaries between work and personal life, the increase in workload and working hours caused by constant connectivity, leading to increased work pressure and a higher likelihood of developing other psychosocial problems. ^14^ While digital workers have the critical skills to lead digital transformation in WFH, their mental health is at risk and must be protected.

There is stigma surrounding mental health in Malaysia, which often leads to patients suffering in silence. Close physical contact and travel was prohibited during the MCO, which made it difficult to seek mental health care. eHealth is able to bridge this gap by using information and communication technology (ICT) to help patients access and manage their health, enable remote communication between doctor and client and integrate data collection and analysis into useful information for more accurate diagnosis. ^13^ eHealth for mental wellness (EHMW), such as Telehealth, can overcome many of the restrictions in seeking health information/care following the MCO, as it eliminates the need for travel and is cost effective. Therefore, higher acceptance of the use of EHMW has great potential to ensure mental wellbeing. As a prerequisite for the effective implementation of EHMW, this research discusses the acceptance of EHMW among digital workers.

The objectives of this research were: 1) to examine the acceptance of EHMW among digital workers in a local premier digital technology institution, and 2) to examine how these talents’ demographic profiles, mental health literacy and workplace wellness influence their acceptance of EHMW.

## Literature review

In addition to physical health, mental health is essential for achieving overall well-being. Therefore, the promotion of mental wellness should also be a priority.

### Application of eHealth for mental wellness

According to Shaw *et al*., thematic analyse interviews with experts involved in health care research revealed that eHealth consists of three general domains: ‘1) Health in our hands is the use of technologies to monitor, track, and inform health status; 2) Interacting for health involves the use of technologies to communicate between stakeholders in health; 3) Data enabling health is the use of technology to collect, manage, and use health data for a more precise diagnostic’. ^13^

The central overlap of three domains indicates the optimum point of eHealth, which is most effective for promoting mental wellness.

### Acceptance of EHMW

Although EHMW is widely accessible and has been shown to be effective, it is still in its early stages, with the acceptability and potential uptake of EHMW still unclear. Previous studies have shown that poor acceptance prevents the uptake of internet-based therapies and results in individuals with mental health problems not seeking professional help. ^3^ The level of interest and real-world uptake remains very low. ^1^ Utilisation and adoption are essential for these technologies to be implemented effectively. ^6^ Furthermore, acceptance of eHealth interventions is highly subjective and often influenced by internal and external factors. Previous research has identified demographic characteristics, such as age and education level, as predictors of eHealth intervention use. ^5^^,^^7^

Knowledge of eHealth has been associated with higher levels of its acceptance. ^5^^,^^13^ Inadequate mental health literacy was identified as a key barrier to seeking help among those with mental health issues. Severity of stress symptoms can be a key determinant of the acceptance of stress-management apps. ^1^ However, there is no clear evidence to link stress to the acceptance of e-mental health. ^2^ The evidence on the influence of eHealth intervention acceptance is inconsistent and differs for various reasons, such as the culture of the study target groups. To address this research gap, we examined the acceptance of EHMW among digital workers in Malaysia and how demographic profiles, mental health literacy and workplace wellness influence acceptance of EHMW. The conceptual framework of this study is depicted in Figure 2.

**Figure 1.**
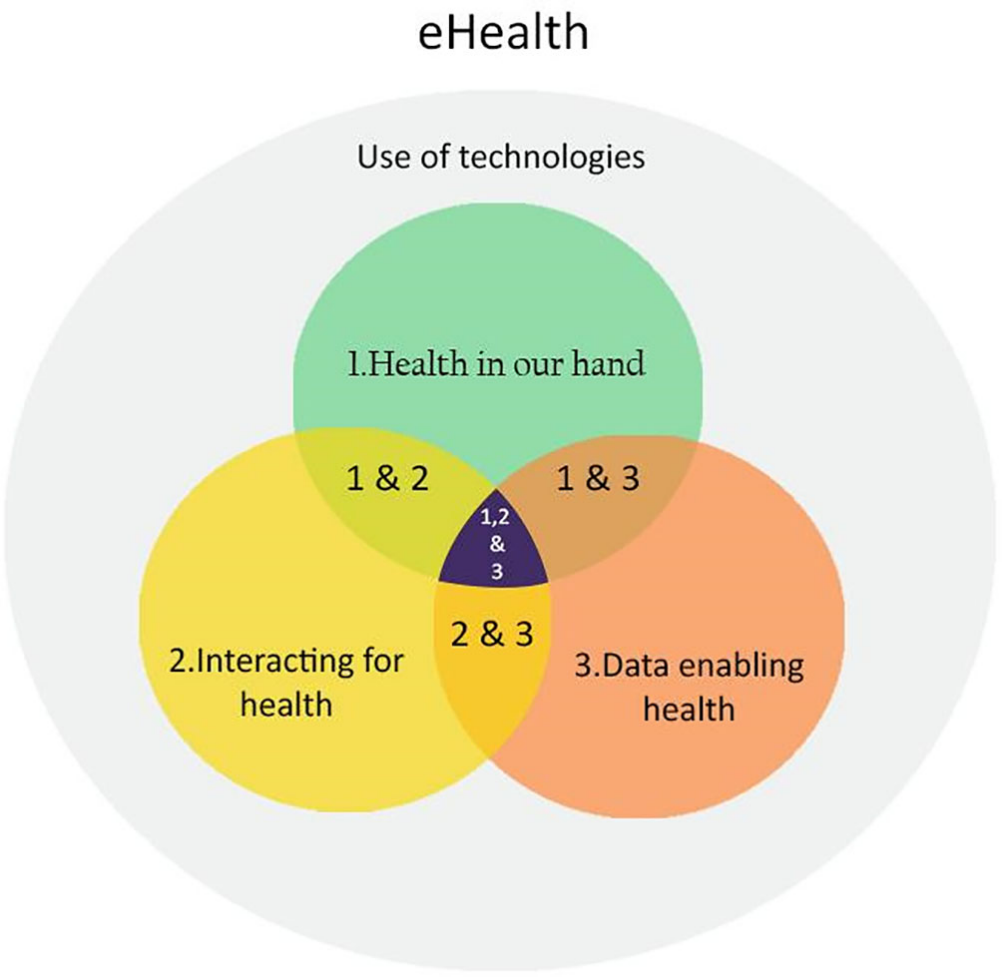
eHealth model by Shaw *et al*. (2017). ^13^

**Figure 2.**
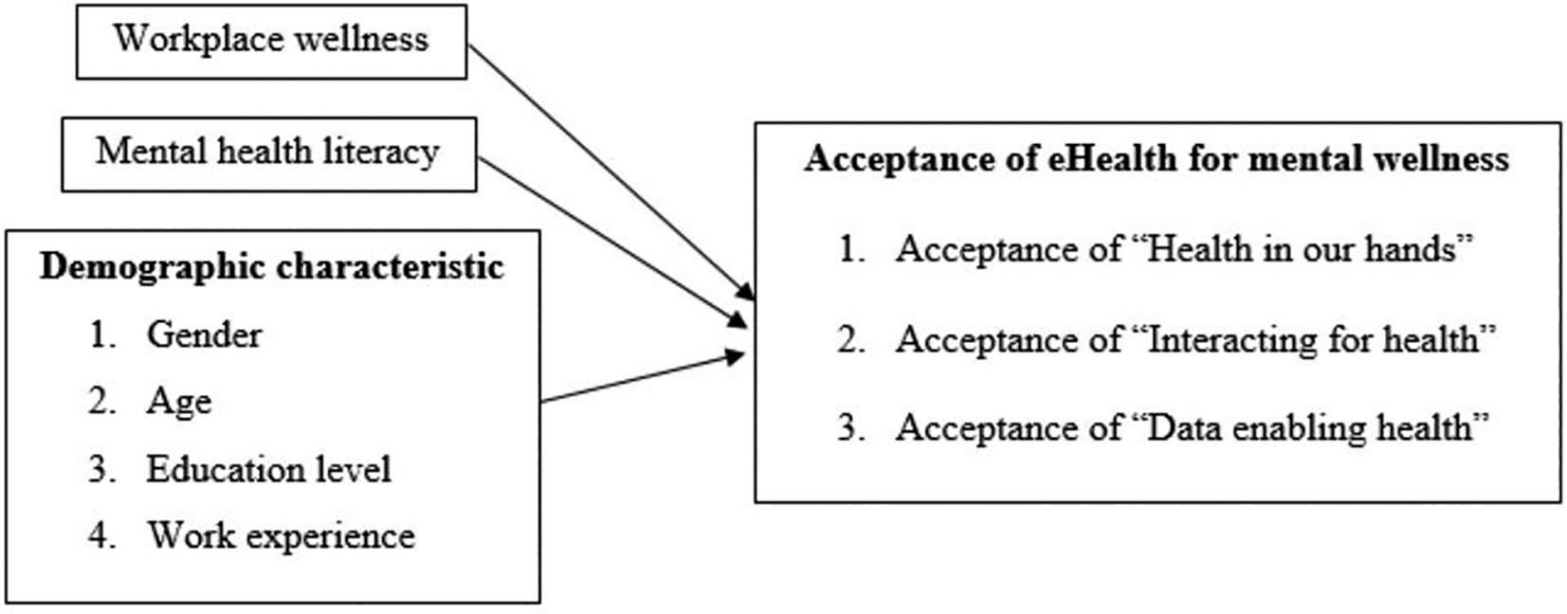
Conceptual framework of the study on acceptance of EHMW.

## Methods

### Study design

This study employed a survey targeted to digital workers from a Premier Digital Tech Institution (PDTI). Data were collected using a questionnaire created through Google Forms that included questions about demographic profiles such as gender, age group, education level, and work experience, as well as acceptance of eHealth. Some of the questions in this questionnaire were adapted from previous research. Following the development of the questionnaire, it was tested for face and content validity by members of the research group who are psychology practitioners, psychology lecturers, and experienced social sciences researchers. The questionnaire was revised in terms of the structure and wording of the questions based on the suggestions. The questionnaire was then sent to four external reviewers for face and content validity testing. These reviewers included lecturers with experience in e-mental health, social science researchers, and data analysis experts. The questionnaire was revised once more to improve the structure and wording of the questions, as well as to remove redundant questions. Finally, the questionnaire was sent to a lecturer who specialises in teaching English language for proofreading. The questionnaire was revised to improve the consistency and accuracy of the questions' grammar, spelling, and punctuation. The survey was conducted online between December 2020 and February 2021. Respondents were recruited via email invitation one month before data collection.

### Data collection

The key variables of this research were measured as follows:

• Mental Health Literacy Scale (MHLS) adapted from the eHealth Literacy Scale (eHEALS) ^6^
• Workplace wellness scale (WWS) adapted from WHO (five-item) Well-Being Questionnaire and ‘Utrecht Work Engagement Scale’ (UWES) ^11^
• Acceptance of EHMW was measured using the three domains of eHealth. ^13^ Items measuring acceptance of ‘health in our hands’ (ACC-HIOH), ‘interacting for health’ (ACC-IFH) and ‘data enabling health’ (ACC-DEH) were adapted from the ‘Unified theory of acceptance and use of technology’ (UTAUT) by Venkatesh *et al*. (2003). Wording of items was adapted into the context of ‘application for mental wellness’

The MHLS consists of five questions with a five-point scale ranging from ‘Strongly disagree’ (1) to ‘Strongly agree’ (5), and a total score ranging between 5 and 25. The WWS consists of five questions with a seven-point scale ranging from ‘At no time’ (0) to ‘All of the time’ (6), and the total score ranging between 0 and 30. The domains ACC-HIOH, ACC-IFH and ACC-DEH each consist of three questions with a five-point scale ranging from ‘Strongly disagree’ (1) to ‘Strongly agree’ (5), and total score ranging between 3 and 15.

Cronbach’s alpha (a) was used to test the item reliability of MHLS-5, WWS, ACC-HIOH, ACC-IFH and ACC-DEH. All scales had acceptable reliability coefficients, with Cronbach’s alpha > 0.7, MHLS-5 = 0.83, WWS = 0.92, ACC-HIOH = 0.83, ACC-IFH = 0.90 and ACC-DEH = 0.89.

### Study population

The study population were employees of a local university that provide digital, computing and multimedia education. It is one out of the 11 premier digital technology universities in Malaysia, where digital workers can be sourced. A purposive sample of 67 employees were selected and invited to participate in an online survey between December 2020 and February 2021. These participants were selected because of their experience of three and more years in teaching IT, computing and multimedia courses or managing computing systems and lab systems.

### Analysis

46 out of 67 respondents responded and participated in this survey. The collected data was exported into Microsoft Excel for data cleaning. Responses that did not provide consent to participate in this survey were removed, leaving 41 valid responses for further analysis. IBM SPSS Statistics Version 23 was used to analyse the data. Descriptive statistics such as mean and standard deviation, independent samples t-test and Pearson’s correlation were used to examine group differences and relationships of data. Because the questionnaire only contains positive coded items, common method bias (CMB) may arise from similar response tendencies caused by similarities in item structure. ^9^ Harman’s single-factor test is used to test CMB, and the results show that the data collected does not contain CMB and is ready for further analysis. ^10^

### Ethics and consent

Ethical approval and clearance for data collection in this study were obtained from Multimedia University (MMU) (Approval number: EA2652021). The questionnaire included an initial option for participants to confirm they consent to participate. Before starting the survey, participants were informed that all identifying information would be kept private, all answers would be collected anonymously and the research report would only present the collective results, and data collected would be held confidentially and used for research purposes only. Only those who responded “Yes” to this question were able to begin the questionnaire.

## Results

**Figure 3.**
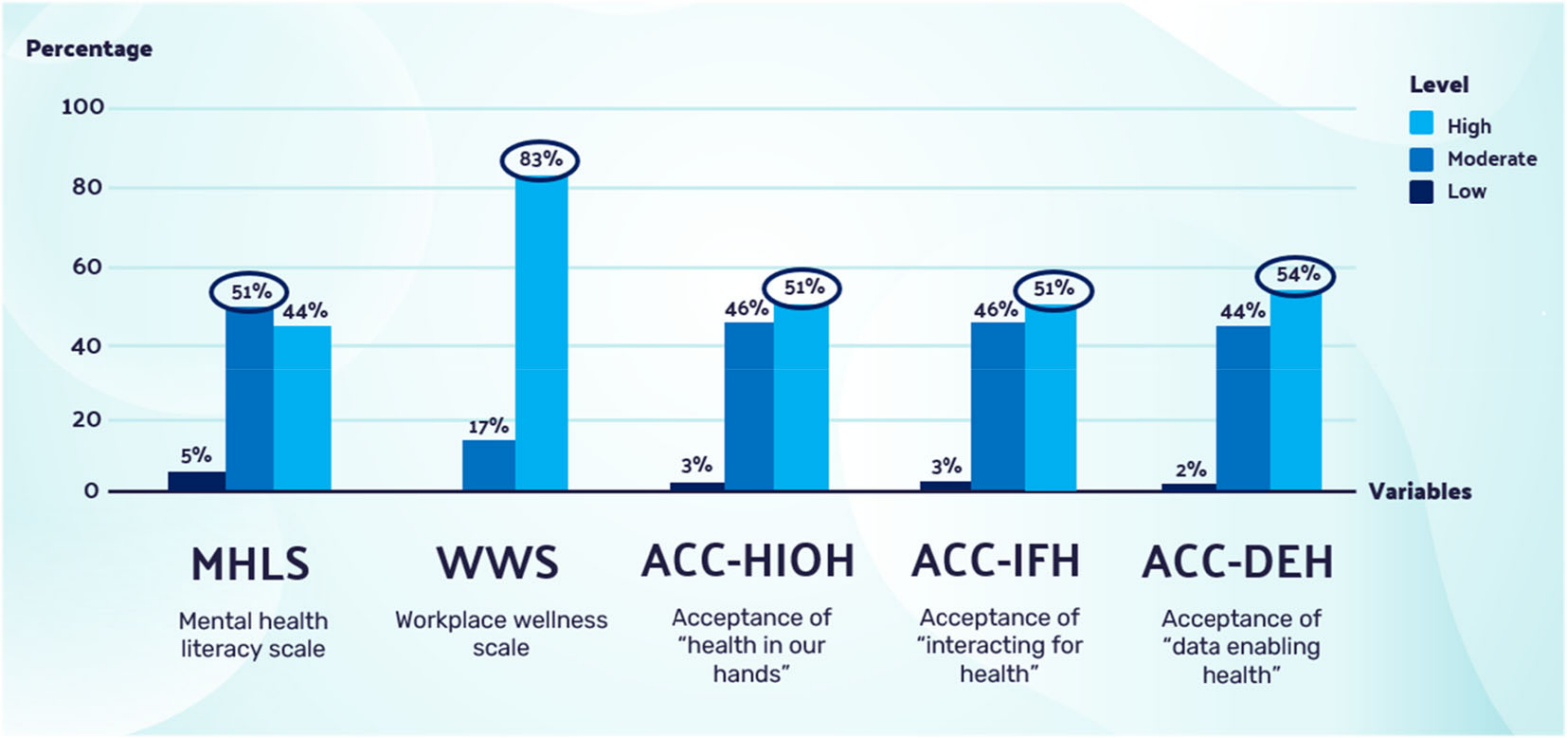
MHLS, WWS, ACC-HIOH, ACC-IFH and ACC-DEH among digital workers. MHLS, WWS, ACC-HIOH, ACC-IFH and ACC-DEH were normally distributed, with skewness between −0.54 to 0.08 and kurtosis between −0.46 to 0.50.

The survey results describe the digital workers’ demographic information, mental health literacy, workplace wellness and acceptance of the EHMW. All responses for the MHLS, WWS, ACC-HIOH, ACC-IFH and ACC-DEH were summarised and averaged to get the mean score, then further categorised into ‘Low’ (1–2.34), ‘Moderate’ (2.35–3.67) and ‘High’ (3.68–5) scores. The WWS was further categorised by the mean score into ‘Low’ (0–1.99), ‘Moderate’ (2–3.99) and ‘High’ (4–6) scores, and the frequency of each score category was calculated. There were 54% (n = 22) females and 46% (n = 19) males; the majority of them, 83% (n = 34), were between the ages of 35 and 54 years old; half of them (n = 21) were Malay and 39% (n = 16) were Chinese, with 5% (n = 2) Indian and 5% (n = 2) other. The majority of respondents, 81% (n = 33), were talents with many years of work experience, ranging from 9–25 years. Most were from Creative Technology and Design (39%, n = 16), followed by Computer Science (20%, n = 8) and IT (17%, n = 7) departments. A total of 44% (n = 18) had a master’s degree, and 39% (n = 16) had a doctorate (PhD). Half of the respondents, 54% (n = 22), had incomes ranging from Ringgit Malaysia (RM)5,880–RM10,959. Most respondents presented with moderate to high levels of mean scores in the MHLS, WWS, ACC-HIOH, ACC-IFH and ACC-DEH, as outlined in Figure 3. The mean score for ACC-IFH (mean = 3.75, SD = 0.68) was the highest compared to ACC-HIOH (mean = 3.69, SD = 0.64) and ACC-DEH (mean = 3.65, SD = 0.66).

An independent samples t-test was used to identify differences between gender, age group and education level and work experience regarding acceptance of the EHMW. There were significant differences in the acceptance of all three eHealth domains for mental wellness between different genders, as depicted in Table 1. For ACC-HIOH, mean scores for males (M = 3.96, SD = 0.63) were higher than females (M = 3.45, SD = 0.56) (t[39] = 2.75, p = 0.009). For ACC-IFH, mean scores for males (M = 4.00, SD = 0.68) were higher than females (M = 3.53, SD = 0.61) (t[39] = 2.33, p = 0.025). For ACC-DEH, mean scores for males (M = 3.93, SD = 0.56) were higher than females (M = 3.41, SD = 0.66) (t[39] = 2.70, p = 0.010).

**Table 1.** Comparisons of mean scores of acceptance of eHealth domains for mental wellness between genders using independent sample t-tests.

Scale item	Male		Female		t(39)	p
	M	SD	M	SD		
ACC-HIOH	3.96	0.63	3.45	0.56	2.75	0.009**
ACC-IFH	4.00	0.68	3.53	0.61	2.33	0.025*
ACC-DEH	3.93	0.56	3.41	0.66	2.70	0.010**

p < 0.05 *, p < 0.01 **, p < 0.001***.

ACC-HIOH = acceptance of ‘health in our hands’; ACC-IFH = acceptance of ‘interacting for health’; ACC-DEH = acceptance of ‘data enabling health’.

There were no significant differences in acceptance of all three eHealth domains for mental wellness between different education levels, as depicted in Table 2. For ACC-HIOH, mean scores for those without a PhD (M = 3.79, SD = 0.59) were slightly higher than for those with a PhD (M = 3.54, SD = 0.70) (t[39] = 1.21, p = 0.24). For ACC-IFH, mean scores for those without a PhD (M = 3.79, SD = 0.64) were slightly higher than for those with a PhD (M = 3.68, SD = 0.75) (t[39] = 0.45, p = 0.65). For ACC-DEH, mean scores for those without a PhD (M = 3.63, SD = 0.63) were slightly lower than for those with a PhD (M = 3.69, SD = 0.72) (t[39] = −0.28, p = 0.78).

**Table 2.** Comparisons of mean scores of acceptance of eHealth domains for mental wellness between education-level groups using independent sample t-tests.

Scale item	Non-PHD		PHD		t(39)	p
	M	SD	M	SD		
ACC-HIOH	3.78	0.59	3.54	0.70	1.21	0.24
ACC-IFH	3.79	0.64	3.69	0.75	0.45	0.65
ACC-DEH	3.63	0.63	3.69	0.72	−0.28	0.78

p < 0.05 *, p < 0.01 **, p < 0.001***.

ACC-HIOH = acceptance of ‘health in our hands’; ACC-IFH = acceptance of ‘interacting for health’; ACC-DEH = acceptance of ‘data enabling health’.

There were no significant differences in acceptance of all three eHealth domains for mental wellness between different age groups, as depicted in Table 3. For ACC-HIOH, mean scores for 25-39-year-olds (group 1; M = 3.86, SD = 0.55) were slightly higher than 40-59-year-olds (group 2; M = 3.60, SD = 0.67) (t[39] = 1.21, p = 0.24). For ACC-IFH, mean scores for age group 1 (M = 3.95, SD = 0.63) were slightly higher than group 2 (M = 3.64, SD = 0.69) (t[39] = 1.41, p = 0.17). For ACC-DEH, mean scores for age group 1 (M = 3.74, SD = 0.68) were slightly higher than group 2 (M = 3.60, SD = 0.66) (t[39] = 0.61, p = 0.55).

**Table 3.** Comparisons of mean scores of acceptance of eHealth domains for mental wellness between age groups using independent sample t-tests.

Scale item	25-39 years old		40-59 years old and above		t(39)	p
	M	SD	M	SD		
ACC-HIOH	3.86	0.55	3.60	0.67	1.21	0.24
ACC-IFH	3.95	0.63	3.64	0.69	1.41	0.17
ACC-DEH	3.74	0.68	3.60	0.66	0.61	0.55

p < 0.05 *, p < 0.01 **, p < 0.001***.

ACC-HIOH = acceptance of ‘health in our hands’; ACC-IFH = acceptance of ‘interacting for health’; ACC-DEH = acceptance of ‘data enabling health’.

There were no significant differences in acceptance of all three eHealth domains for mental wellness between different work experience groups, as depicted in Table 4. For ACC-HIOH, mean scores for those with 3-15 years of work experience (group 1; M = 3.74, SD = 0.61) were slightly higher than for those with 16-25 years of work experience (group 2; M = 3.65, SD = 0.67) (t[39] = 0.42, p = 0.68). For ACC-IFH, mean scores for group 1 (M = 3.70, SD = 0.55) were slightly lower than group 2 (M = 3.79, SD = 0.78) (t[39] = −0.40, p = 0.69). For ACC-DEH, mean scores for group 1 (M = 3.74, SD = 0.59) were slightly higher than group 2 (M = 3.58, SD = 0.72) (t[39] = 0.77, p = 0.44).

**Table 4.** Comparisons of mean scores of acceptance of eHealth domains for mental wellness between work experience groups using independent sample t-tests.

Scale item	3-15 years		16-25 years and above		t(39)	p
	M	SD	M	SD		
ACC-HIOH	3.74	0.61	3.65	0.67	0.42	0.68
ACC-IFH	3.70	0.55	3.79	0.78	−0.40	0.69
ACC-DEH	3.74	0.59	3.58	0.72	0.77	0.44

p < 0.05 *, p < 0.01 **, p < 0.001***.

ACC-HIOH = acceptance of ‘health in our hands’; ACC-IFH = acceptance of ‘interacting for health’; ACC-DEH = acceptance of ‘data enabling health’.

Pearson’s correlation (r) analysis was conducted to analyse the bivariate correlations of the MHLS, WWS, ACC-HIOH, ACC-IFH and ACC-DEH, and significant correlations were found. MHLS and WWS were negatively correlated (Pearson’s r (41) = −0.33, p = 0.036). ACC-HIOH was positively correlated with ACC-IFH (Pearson’s r(41) = 0.78, p <0.001) and ACC-DEH (Pearson’s r(41) = 0.70, p <0.001). ACC-IFH was positively correlated with ACC-DEH (Pearson’s r(41) = 0.71, p <0.001). However, there were no significant correlations between MHLS and ACC-HIOH, ACC-IFH or ACC-DEH, and no significant correlations between WWS and ACC-HIOH, ACC-IFH or ACC-DEH, as depicted in Table 5.

**Table 5.** Correlations between the MHLS, WWS, ACC-HIOH, ACC-IFH and ACC-DEH.

Scale item	1	2	3	4	5
1. MHLS	—	−0.33*	−0.025	0.022	0.094
2. WWS	−0.33*	—	0.13	0.047	−0.014
3. ACC-HIOH	−0.025	0.13	—	0.78**	0.70**
4. ACC-IFH	0.022	0.047	0.78*	—	0.71**
5. ACC-DEH	0.094	−0.014	0.70**	0.71	—

Correlation is significant at the 0.05 level (two-tailed)* and the 0.01 level (two-tailed)**.

MHLS = Mental Health Literacy Scale; WSS = workplace wellness scale; ACC-HIOH = acceptance of ‘health in our hands’; ACC-IFH = acceptance of ‘interacting for health’; ACC-DEH = acceptance of ‘data enabling health’.

## Discussion

This research provided information on the acceptance of EHMW among digital workers in Malaysia and showed moderate to high acceptance of EHMW. The purposively selected respondents were digitally experienced talents, who seem to have high acceptance, similar to previous findings showing that frequent internet users are more willing to use the internet for mental health purposes. ^2^ A deeper understanding of EHMW acceptance was examined by adopting the three eHealth domains, which showed that acceptance of ‘interaction for health’ (IFH) was highest among the other eHealth domains. This is consistent with previous studies showing that IFH applications, such as Tele-mental Health, that use communication technologies to deliver mental health care remotely are widely accepted in mental health treatment, especially during MCOs, as they eliminate the need for travel while maintaining the quality of health care, which is cost- effective. ^15^

The three domains of eHealth showed strong correlations, implying that acceptance of any one of the eHealth domains may be a strong predictor of acceptance of the other domains. In this study, there was a significant difference in the acceptance of EHMW between genders, with males having a higher acceptance rate of EHMW than females, which is supported by previous findings indicating that males with a more technology-friendly orientation have a higher acceptance rate of e-mental health ^6^ and implies that it is important to promote the acceptance of EHMW among female digital workers. There was no significant difference in the acceptance of EHMW among different age groups, education levels and level of work experience. Mental health literacy and wellness at work did not have a strong influence on EHMW acceptance, reflecting the findings of previous research, which indicated that EHMW literacy was only indirectly related to EHMW acceptance ^6^ and that perceived stress was not a meaningful predictor of acceptance. ^2^

## Conclusion

The prevention of mental health problems is important during the COVID-19 crisis, and EHMW has played an important role in providing mental health-related support during the MCO. Nevertheless, it is predicted that social distancing measures coupled with awareness among policy and decision makers in the context of the pandemic will lead to significant attitudinal and behavioural change and result in greater long-term acceptance of EHMW. As the acceptance of EHMW in this paper was researched using a small survey of digital workers, it was not possible to fully examine acceptance and the determinants that might influence acceptance of EHMW among digital workers in Malaysia. A larger study by this research group is currently underway to capture further aspects regarding the determining factors of EHMW acceptance.

## Data availability

### Underlying data

DANS-EASY: Exploring acceptance of the eHealth model for mental wellness among digital workers. https://doi.org/10.17026/dans-xwz-5x6s ^12^

This project contains the following underlying data:

- Dataset.xlsx (This file consists all 41 responses that were collected from respondents that participated in the survey for this study)

### Extended data

This project contains the following extended data:

- Survey Questionnaire_EN.pdf (Respondents were required to answer this questionnaire to participate in the survey)

Data are available under the terms of the Creative Commons Zero “No rights reserved” data waiver (CC0 1.0 Public domain dedication).
