# Supplementary material for: A quantitative study exploring the acceptance of the eHealth model for mental wellness among digital workers
Source: F1000Res. 2022 Jun 9;11:111. Originally published 2022 Jan 28. [Version 2] doi: 10.12688/f1000research.73482.2 (PMC9214265; doi:10.12688/f1000research.73482.2)
Supplement: Peer review history

# Peer review: Reviewer response for version 2

**DOI:** 10.5256/f1000research.134083.r140203

**Published:** 2022-06-21

**Authors:** Karin Ahlin

**Affiliations:** 1 Computer and System Sciences, Mid Sweden University, Sundsvall, Sweden

Following the feedback from reviewers, the authors have addressed all the comments accordingly and added new information in the new version of this paper. This added information can be found in Sections "Introduction", "Methods", and "Conclusion".

Is the work clearly and accurately presented and does it cite the current literature?

Yes

If applicable, is the statistical analysis and its interpretation appropriate?

Yes

Are all the source data underlying the results available to ensure full reproducibility?

Yes

Is the study design appropriate and is the work technically sound?

Yes

Are the conclusions drawn adequately supported by the results?

Yes

Are sufficient details of methods and analysis provided to allow replication by others?

Partly

Reviewer Expertise:

e-health

I confirm that I have read this submission and believe that I have an appropriate level of expertise to confirm that it is of an acceptable scientific standard.

# Peer review: Reviewer response for version 2

**DOI:** 10.5256/f1000research.134083.r140204

**Published:** 2022-06-10

**Authors:** Eugene Boon Yau Koh

**Affiliations:** 1 Department of Psychiatry, Faculty of Medicine and Health Sciences, Universiti Putra Malaysia, Serdang, Malaysia

No further comments.

Is the work clearly and accurately presented and does it cite the current literature?

Yes

If applicable, is the statistical analysis and its interpretation appropriate?

Partly

Are all the source data underlying the results available to ensure full reproducibility?

Yes

Is the study design appropriate and is the work technically sound?

Yes

Are the conclusions drawn adequately supported by the results?

Yes

Are sufficient details of methods and analysis provided to allow replication by others?

Yes

Reviewer Expertise:

psychiatry, mental health, digital mental health

I confirm that I have read this submission and believe that I have an appropriate level of expertise to confirm that it is of an acceptable scientific standard.

# Peer review: Reviewer response for version 1

**DOI:** 10.5256/f1000research.77136.r129667

**Published:** 2022-05-09

**Authors:** Karin Ahlin

**Affiliations:** 1 Computer and System Sciences, Mid Sweden University, Sundsvall, Sweden

The study is of interest and should be tested in other work groups or other contexts. The interesting part of the study is the combination of various aspects on accepting e-health and the relation to the pandemic situation with the working-from-home situation.

 What I want you to consider is to describe why you have started by using this group of people. Why are they of interest as a focal point? Is it related to easy access or any other specific factor? I also would like to know how you want to continue with other groups, declaring a step-wise declaration on how to move on with them. This would make the study of further interest, declaring how scale it. I would also like to have more information about the group, i.e., in the introduction or even in the article’s headline.

Is the work clearly and accurately presented and does it cite the current literature?

Yes

If applicable, is the statistical analysis and its interpretation appropriate?

Yes

Are all the source data underlying the results available to ensure full reproducibility?

Yes

Is the study design appropriate and is the work technically sound?

Yes

Are the conclusions drawn adequately supported by the results?

Yes

Are sufficient details of methods and analysis provided to allow replication by others?

Partly

Reviewer Expertise:

e-health

I confirm that I have read this submission and believe that I have an appropriate level of expertise to confirm that it is of an acceptable scientific standard, however I have significant reservations, as outlined above.

# Response

**Published:** 2022-05-15

**Authors:** Choon Hong Tan

**Affiliations:** Multimedia University, Malaysia

Thank you for your thoughtful and highly constructive comments and efforts towards improving our manuscript. In the following, we highlight your general concerns by addressing to each comments.

**The study is of interest and should be tested in other work groups or other contexts.**

 Future study will be addressed to larger group of digital workers from various digital and ICT related industry/firms/institutions. This larger study has been conducted and is in the process of data analysis and reporting. Further planning of future studies for other high stress working groups, such as blue collar workers involved in electronics manufacturing organizations who are exposed to high work stress; young trainee doctors who are still conducting housemanship procedures in Malaysia’s government hospitals that experience high burnout rate due to huge workload, and huge mental burden due to workplace violence and bullying. The issue of high prevalence of workplace bullying among young doctors was recently brought to attention following a shocking and sad incident of death of a young doctors which is claimed to be the result of bullying and harassment. The study should also be addressed to these group of workers in order to reveal more findings which would help the future implementation of eHealth to be more relevant and beneficial to these groups of workers who are in need of these eHealth innovations.

**What I want you to consider is to describe why you have started by using this group of people. Why are they of interest as a focal point?**

 The study implement purposive sampling from a chosen multimedia and ICT based higher education institution that offer mostly ICT, computing and multimedia related academic programmes. This group of users / respondents were chosen mainly because they have the experience, background, and technical knowledge in the areas of ICT, computing and multimedia, and are often works with digital technologies to carry out their daily task.

 The target respondents include, 1) in-house system developer, system admin who are under the Technical and ICT Division of the institution, 2) an academician group who are teaching in multimedia and ICT field. These groups of the respondents have more exposure to digital technologies and have been expecting (more ready) to have higher digital literacy and acceptance for new technologies such as e-mental health. Another reason is due to higher education industry in Malaysia is highly competitive and greatly affected by the COVID-19 pandemic. The shifting of workstyle to a new normal, especially in teaching online, and many other stressful situations (such as the increase of student intakes) due to pandemic situation, has affected their mental wellness. Their experience during this period also have made them as an important stakeholder which needs to be a targeted respondents of this study.

**Is it related to easy access or any other specific factor?**

 This research was conducted mainly as purposive sampling (of choosing digital workers), influence partly by the easy access of the sample. This is because the second and the third author is close to the targeted respondent group, and they were able to assist in data collection in a tight schedule given under this study.

**I also would like to know how you want to continue with other groups, declaring a step-wise declaration on how to move on with them. This would make the study of further interest, declaring how scale it.**

 After this study was concluded, another study was conducted to reach out to more digital workers from various digital and ICT related industry/firms and ICT divisions/department from various companies. This study has started and currently in the analysis and reporting stage.

 Future study also planned to reach out to other high stress worker groups such as blue collar workers involved in electronics manufacturing organizations; young trainee doctor who are still conducting housemanship procedures in Malaysia’s government hospitals, and to different workplaces, or different employers who are concerned about their employees’ wellbeing and subsequently promoting health in their workplaces.

 These target groups are chosen because they are familiar with the concept of workplace wellness, and therefore their inputs will be highly beneficial for the development of knowledge in this field.

 Lastly, a more comprehensive research would need to be participatory in nature by including different stakeholders (developers, health care providers, and users) in a more formal and structured program. The program can involve various processes of developing, implementing, and evaluating eHealth for mental wellness and to increase the likelihood of the intervention to be appeal, and to be accepted and meet the users’ need.

 An innovation’s full potential can only be exploited for better wellness when it is well accepted and is being used for a long period of time.

**I would also like to have more information about the group, i.e., in the introduction or even in the article’s headline.**

 More information of the target group will be included in the introduction of the new version of this paper after revision is completed.

# Peer review: Reviewer response for version 1

**DOI:** 10.5256/f1000research.77136.r124565

**Published:** 2022-03-18

**Authors:** Eugene Boon Yau Koh

**Affiliations:** 1 Department of Psychiatry, Faculty of Medicine and Health Sciences, Universiti Putra Malaysia, Serdang, Malaysia

This is a fundamental study that explores the acceptance of the eHealth model for mental health. The study aimed to explore the association of acceptance with participants demographic data, mental health literacy and workplace wellness. They found that participants were agreeable with the idea of eHealth for mental health. They also found that the acceptance level was the highest for the application domain of "interacting for health", with male respondents more likely to accept the use of eHealth in Mental Health.

 The study design and data collection appear to be sound. However, there are other confounding factors such as internet access availability, gender bias in workplace (if relevant in sample population), salary gap and other external factors that can have a huge impact on the current study result. These factors should be briefly addressed by the authors to present a more holistic description of the results.

 As this is a fundamental study, I believed that the results presented, while limited in interpretation, provides an early view on how eHealth can be associated within the field of mental health. This study serve as the stepping stone so that more robust study methodology should be explored in the future.

Is the work clearly and accurately presented and does it cite the current literature?

Yes

If applicable, is the statistical analysis and its interpretation appropriate?

Partly

Are all the source data underlying the results available to ensure full reproducibility?

Yes

Is the study design appropriate and is the work technically sound?

Yes

Are the conclusions drawn adequately supported by the results?

Yes

Are sufficient details of methods and analysis provided to allow replication by others?

Yes

Reviewer Expertise:

psychiatry, mental health, digital mental health

I confirm that I have read this submission and believe that I have an appropriate level of expertise to confirm that it is of an acceptable scientific standard.

# Response

**Published:** 2022-03-20

**Authors:** Choon Hong Tan

**Affiliations:** Multimedia University, Malaysia

Dear Eugene Boon Yau Koh,

 Thank you very much for your reviews.
